# Breast cancer in the era of generative artificial intelligence: assistant tools for clinical doctors based on ChatGPT

**DOI:** 10.1097/JS9.0000000000001597

**Published:** 2024-05-08

**Authors:** Yue Zheng, Xu Sun, Kai Kang, Ailin Zhao, Yijun Wu

**Affiliations:** a Division of Thoracic Tumor Multimodality Treatment, Cancer Center, West China Hospital, Sichuan University; b Laboratory of Clinical Cell Therapy, West China Hospital, Sichuan University; c Department of Hematology, West China Hospital, Sichuan University, Chengdu, Sichuan, People’s Republic of China

*Dear Editor,*

Large Language Models (LLMs) have garnered widespread attention in the field of artificial intelligence (AI) due to their outstanding contextual understanding and responsiveness^1^. However, the potential of LLMs in the diagnosis, treatment, and care of breast cancer remains insufficiently explored. The study conducted by Deng *et al*.^2^ represents a significant step forward in understanding the potential application of LLMs in the clinical context of breast cancer. The comparison between ChatGPT-3.5, ChatGPT-4.0, and Claude2 sheds light on the varying capabilities of these models and underscores the importance of domain-specific optimization in advancing the utility of AI in critical medical scenarios.

After carefully reading the article, we have several questions for the authors and fellow researchers to gain a more comprehensive understanding of the paper.

Firstly, regarding the heterogeneity issue in LLMs responses. The GPT series models are trained on vast amounts of internet text data, implying that their knowledge and language patterns are built upon the foundation of current internet information^3^. However, the medical field is a constantly updating and evolving domain, with new research findings, clinical guidelines, and medical technologies emerging continuously, resulting in a rapid pace of knowledge updates in medicine. ChatGPT-3.5, ChatGPT-4.0, and Claude2 being models released at different points in time, would have differences in their training data and knowledge bases. For example, ChatGPT-3.5 might have been trained in earlier stages, potentially leaning toward earlier medical research findings and clinical practices. As time progresses, ChatGPT-4.0 and Claude2 may incorporate updated and more extensive data and knowledge, better reflecting current medical practices and the latest research findings. However, even models released at the same time may have differences in the knowledge they encompass^4^. This depends on factors such as the sources of training data, the size and diversity of datasets, and optimization techniques of training algorithms. In addition to the temporal aspect of the models themselves, the pace of medical information updates needs consideration. Medical knowledge updates typically occur on a yearly basis or even shorter timeframes. New clinical trial results, drug efficacy data, disease diagnostic criteria, etc., can change rapidly, affecting the accuracy and timeliness of model responses. Therefore, we need to recognize that models released at different times may have different knowledge bases and timeliness, potentially leading to different answers when addressing specific medical questions. When using these models for medical decision-making or providing medical advice, caution is warranted, and a comprehensive consideration of the latest clinical practices and research findings is necessary.

Furthermore, regarding the five expert panels evaluating LLM performance, it is essential to determine whether they are from different centers, regions, or even countries, as experiences in medical practice may vary across different centers or regions. When evaluating LLM performance, considering the diversity and representativeness of the evaluation team is crucial. Experiences in medical practice may vary significantly across different centers, regions, or even countries, primarily influenced by healthcare resources, cultural backgrounds, patient characteristics, and healthcare systems. Thus, recruiting evaluation expert teams from multiple regions, centers, or countries is necessary to ensure the comprehensiveness and representativeness of evaluation results^5^. Firstly, experts from different regions can bring diverse medical practice experiences and cultural backgrounds. Healthcare systems, diagnostic and treatment standards, and resource allocations may vary greatly across different regions, leading to differences in how doctors handle the same cases. Therefore, including experts from different regions can provide broader perspectives and experiences, aiding in evaluating the applicability and effectiveness of LLMs in different cultural and medical environments. Secondly, experts from different centers can represent different clinical practices and research teams. Different medical centers may have distinct clinical features, research focuses, and personnel compositions, all of which can influence experts’ perspectives and focal points when evaluating LLM performance. By involving experts from multiple centers, the universality and credibility of evaluation results can be ensured. Lastly, considering that medical guidelines, while covering fundamental cognition, often require support from personal experience in actual clinical work, experts in the evaluation team should possess rich clinical experience and practical abilities capable of assessing LLM performance from the perspective of practical clinical application. Additionally, biases such as personal experience preferences, affiliations, or healthcare system biases should be noted during the evaluation process, with corresponding measures taken for control and balance. Therefore, when evaluating LLM performance, the diversity and representativeness of the evaluation team, especially the combination of experts from different regions and centers, should be considered. This helps ensure the comprehensiveness, objectivity, and credibility of evaluation results, providing more reliable support and guidance for the application of LLMs in clinical practice.

In the realm of breast cancer, AI and LLMs play a multifaceted role, encompassing various applications aimed at enhancing screening, diagnosis, treatment, and management practices. Through the utilization of machine learning and deep learning algorithms, AI facilitates early screening and diagnosis by developing tools capable of analyzing diverse imaging data, including mammograms, ultrasound images, and magnetic resonance imaging. These tools assist healthcare professionals in identifying potential anomalies and tumors, thereby improving the chances of early detection and intervention. Moreover, AI leverages patient-specific genomic data and clinical information to enable personalized therapy recommendations. By predicting patient responses to specific drugs, forecasting the risk of disease progression, and aiding in the selection of optimal treatment strategies, AI empowers clinicians to tailor treatment plans to individual patients, maximizing efficacy and minimizing adverse effects. Furthermore, AI supports treatment decision-making by providing clinicians with decision support systems based on extensive clinical data and research findings. These systems offer objective recommendations and references, aiding clinicians in making informed and accurate decisions regarding treatment and surgical plans. In addition to clinical applications, AI facilitates remote monitoring and management of breast cancer patients through smart devices and remote medical platforms. By continuously monitoring physiological parameters and activity levels through wearable devices and offering regular remote medical consultations, AI enables proactive and personalized care delivery, even outside traditional healthcare settings. Finally, AI contributes to advancing scientific research in breast cancer by aiding researchers in analyzing large-scale clinical data and biological information. By uncovering potential disease mechanisms, diagnostic biomarkers, and therapeutic targets, AI accelerates progress in both scientific understanding and clinical practice in the field of breast cancer. Overall, the integration of AI holds immense promise for revolutionizing breast cancer care by improving early detection, personalizing treatment approaches, supporting clinical decision-making, enabling remote monitoring, and driving scientific innovation.

Moving forward, further research is warranted to explore the potential of AI as well as LLMs in addressing specific clinical challenges and to develop robust frameworks for integrating these models into healthcare workflows effectively. By addressing the identified concerns and leveraging the strengths of LLMs in conjunction with clinical expertise, we can harness the full potential of AI to enhance diagnosis, treatment, and care delivery for patients with breast cancer and beyond.

## Ethical approval

Not applicable.

## Sources of funding

This work was supported by the Postdoctoral Fellowship Program of CPSF (No. GZB20230481), National Natural Science Foundation of China (No. 82303773, No. 82204490, No. 82303772), Natural Science Foundation of Sichuan Province (No. 2023NSFSC1885), and Key Research and Development Program of Sichuan Province (No. 23ZDYF2836).

## Author contribution

All authors read and approved the final version of the manuscript.

## Conflicts of interest disclosure

The authors declare that they have no conflicts of interest.

## Research registration unique identifying number (UIN)

Not applicable.

## Guarantor

All the authors of this paper accept full responsibility for the work and/or the conduct of the study, have access to the data, and control the decision to publish.

## Data availability statement

No primary data were generated and reported in this manuscript. Therefore, data have not become available to any academic repository.
